# Supplementary material for: Machine Learning Accelerates Crystallization for Structure Determination
Source: Angew Chem Int Ed Engl. 2026 May 4;65(25):e1218503. doi: 10.1002/anie.1218503 (PMC13266958; doi:10.1002/anie.1218503)

checkCIF/PLATON report

Structure factors have been supplied for datablock(s) lcz028

THIS REPORT IS FOR GUIDANCE ONLY. IF USED AS PART OF A REVIEW PROCEDURE FOR PUBLICATION, IT SHOULD NOT REPLACE THE EXPERTISE OF AN EXPERIENCED CRYSTALLOGRAPHIC REFEREE.

No syntax errors found. CIF dictionary Interpreting this report

Datablock: lcz028

Bond precision: C-C = 0.0194 A Wavelength=1.54184

Cell:	a=8.4700(3)	b=8.5735(3)	c=12.4840(5)
	alpha=74.978(3)	beta=70.595(3)	gamma=87.087(3)
Temperature:	100 K		

	Calculated	Reported
Volume	825.22(6)	825.22(6)
Space group	P 1	P 1
Hall group	P 1	P 1
Moiety formula	C26 H15 Ag3 F18 N6 O3	C15 H3 Ag3 F18 N6, C11 H12 O3
Sum formula	C26 H15 Ag3 F18 N6 O3	C26 H15 Ag3 F18 N6 O3
Mr	1125.05	1125.05
Dx,g cm-3	2.264	2.264
Z	1	1
Mu (mm-1)	15.494	15.494
F000	540.0	540.0
F000'	542.62	
h,k,lmax	10,10,15	10,10,15
Nref	7048[3524]	3965
Tmin,Tmax	0.061,0.045	0.248,1.000
Tmin'	0.004	

Correction method= # Reported T Limits: Tmin=0.248 Tmax=1.000
AbsCorr = MULTI-SCAN

Data completeness= 1.13/0.56 Theta(max)= 77.905

R(reflections)= 0.0517(3929) wR2(reflections)= 0.1388(3965)

S = 1.049 Npar= 506

The following ALERTS were generated. Each ALERT has the format
test-name_ALERT_alert-type_alert-level.
Click on the hyperlinks for more details of the test.

Alert level B

```
PLAT915_ALERT_3_B No Flack x Check Done: Low Friedel Pair Coverage        18 %
```

Author Response: Due to insufficient data. The compound crystallizes in the chiral space group, but no chiral ligand is used, and the complex is no longer chiral.

Alert level C

```
PLAT042_ALERT_1_C Calc. and Reported MoietyFormula Strings  Differ     Please Check
              Calc: C26 H15 Ag3 F18 N6 O3
              Rep.: C15 H3 Ag3 F18 N6, C11 H12 O3
PLAT090_ALERT_3_C Poor Data / Parameter Ratio (Zmax > 18) ........       6.61 Note
PLAT213_ALERT_2_C Atom F00P            has ADP max/min Ratio .....        3.3 prolat
PLAT213_ALERT_2_C Atom N00J            has ADP max/min Ratio .....        3.1 oblate
PLAT342_ALERT_3_C Low Bond Precision on  C-C Bonds ...............    0.01941 Ang.
PLAT911_ALERT_3_C Missing FCF Refl Between Thmin & STh/L=    0.600         18 Report
                2 10  2,   8 -5  3,   4  0  3,  10  1  3,  -6  7  4,  -5  8  4,
               -5  7  5,  -5  8  5,  -5  7  6,   7  5  8,   4 -6  9,  -4 -3  9,
                0  0  9,  -3 -3 10,  -2  6 10,  -2  6 11,  -2  7 11,   3  6 14,
PLAT971_ALERT_2_C Check Calcd Resid. Dens.  1.06Ang From Ag01          2.28 eA-3
PLAT971_ALERT_2_C Check Calcd Resid. Dens.  1.00Ang From N00Q          2.17 eA-3
PLAT971_ALERT_2_C Check Calcd Resid. Dens.  0.91Ang From Ag02          2.11 eA-3
PLAT971_ALERT_2_C Check Calcd Resid. Dens.  1.10Ang From Ag02          2.10 eA-3
PLAT971_ALERT_2_C Check Calcd Resid. Dens.  1.10Ang From Ag01          2.06 eA-3
PLAT971_ALERT_2_C Check Calcd Resid. Dens.  1.02Ang From N00J          1.96 eA-3
PLAT971_ALERT_2_C Check Calcd Resid. Dens.  1.02Ang From Ag02          1.60 eA-3
```

Alert level G

```
PLAT002_ALERT_2_G Number of Distance or Angle Restraints on AtSite          2 Note
PLAT003_ALERT_2_G Number of Uiso or U(i,j)  Restrained non-H-Atoms          5 Report
PLAT072_ALERT_2_G SHELXL First  Parameter in WGHT  Unusually Large       0.12 Report
PLAT154_ALERT_1_G The s.u.'s on the Cell Angles are Equal ..(Note)      0.003 Degree
PLAT172_ALERT_4_G The CIF-Embedded .res File Contains DFIX Records          1 Report
PLAT186_ALERT_4_G The CIF-Embedded .res File Contains ISOR Records          2 Report
PLAT242_ALERT_2_G Low    'MainMol' Ueq as Compared to Neighbors of       C00V Check
PLAT242_ALERT_2_G Low    'MainMol' Ueq as Compared to Neighbors of       C011 Check
PLAT242_ALERT_2_G Low    'MainMol' Ueq as Compared to Neighbors of       C014 Check
PLAT242_ALERT_2_G Low    'MainMol' Ueq as Compared to Neighbors of       C016 Check
```

```
PLAT242_ALERT_2_G Low    'MainMol' Ueq as Compared to Neighbors of       C01C Check
PLAT242_ALERT_2_G Low    'MainMol' Ueq as Compared to Neighbors of       C01D Check
PLAT720_ALERT_4_G Number of Unusual/Non-Standard Labels ..........         71 Note
              Ag01    Ag02    Ag03    F004    F005    F006    F007    F008
              F009    F00A    F00B    F00C    F00D    F00E    F00F    N00G
              F00H    O00I    N00J    N00K    O00L    O00M    F00N    F00O
              F00P    N00Q    N00R    N00S    C00T    H00T    F00U    C00V
              C00W    H00W    F00X    C00Y    C00Z    C010    H010    C011
              C012    C013    H013    C014    C015    C016    C017    C018
              C019    H019    C01A    C01B    C01C    C01D    C01E    H01A
              H01B    C01F    C01G    H01G    C01H    C01I    H01C    H01D
              C01J    H01E    H01F    C01K    H01H    H01I    H01J
PLAT860_ALERT_3_G Number of Least-Squares Restraints .............         34 Note
PLAT883_ALERT_1_G Absent Datum for _atom_sites_solution_primary ..     Please Do !
PLAT912_ALERT_4_G Missing # of FCF Reflections Above STh/L=  0.600        159 Note
PLAT933_ALERT_2_G Number of HKL-OMIT Records in Embedded .res File          1 Note
                8 -6  1,
PLAT941_ALERT_3_G Average HKL Measurement Multiplicity ...........        2.0 Low
PLAT969_ALERT_5_G The 'Henn et al.' R-Factor-gap value ...........      4.999 Note
              Predicted wR2: Based on SigI**2  2.78 or SHELX Weight 13.23
PLAT978_ALERT_2_G Number C-C Bonds with Positive Residual Density.          0 Info
```

```
   0 ALERT level A = Most likely a serious problem - resolve or explain
   1 ALERT level B = A potentially serious problem, consider carefully
  13 ALERT level C = Check. Ensure it is not caused by an omission or oversight
  20 ALERT level G = General information/check it is not something unexpected

   3 ALERT type 1 CIF construction/syntax error, inconsistent or missing data
  20 ALERT type 2 Indicator that the structure model may be wrong or deficient
   6 ALERT type 3 Indicator that the structure quality may be low
   4 ALERT type 4 Improvement, methodology, query or suggestion
   1 ALERT type 5 Informative message, check
```

It is advisable to attempt to resolve as many as possible of the alerts in all categories. Often the minor alerts point to easily fixed oversights, errors and omissions in your CIF or refinement strategy, so attention to these fine details can be worthwhile. It is up to the individual to critically assess their own results and, if necessary, seek expert advice.

PLATON version of 17/09/2025; check.def file version of 17/09/2025

---

duplicate check

No duplication found

Datablock lcz028 - ellipsoid plot

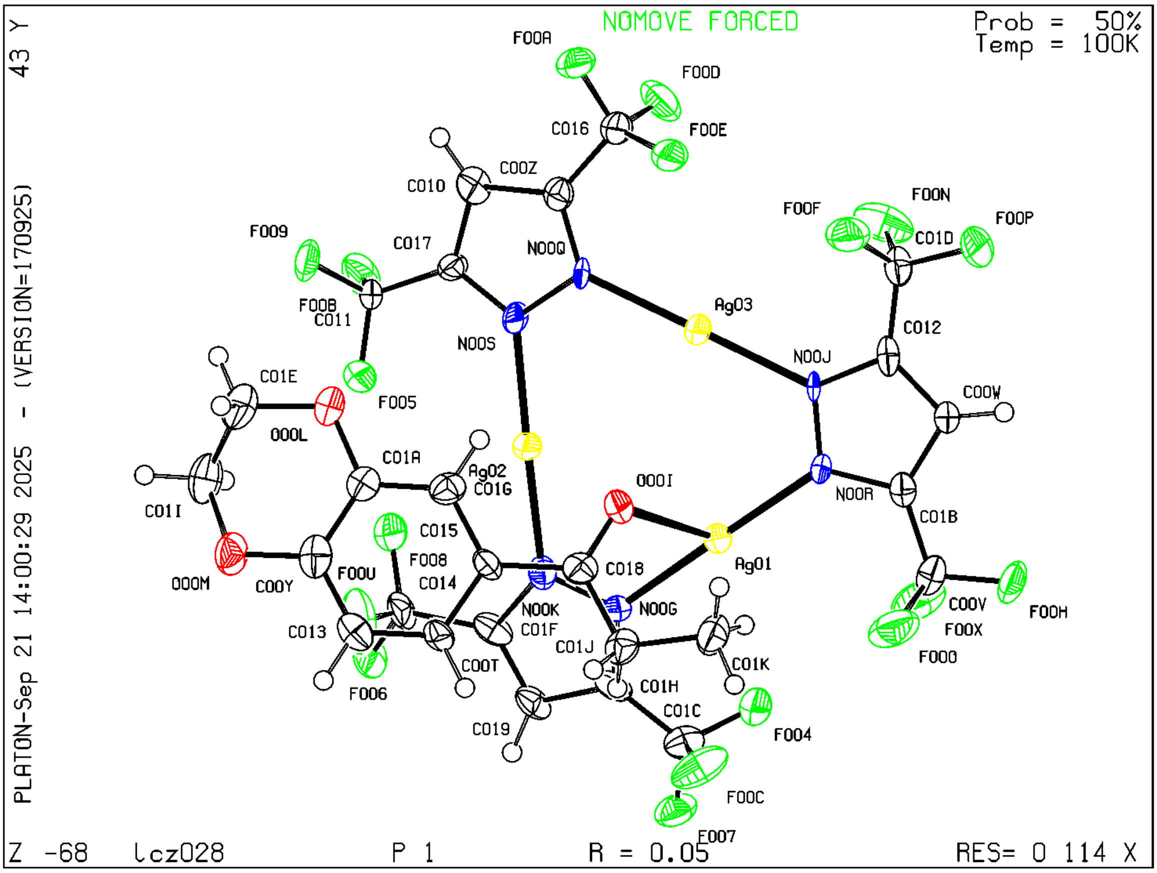

Supplement: Supplementary file 2 — Supporting File 2: anie72462‐sup‐0002‐DataFile.zip. [file ANIE-65-e1218503-s002.zip › supplementary_materials_crystallographic_data submit.zip!/cif_and_checkcif(1-114)/Ag3Pz3_28/Ag3Pz3_28_checkcif.pdf]
